# Molecular SARS-CoV-2 surveillance in Bavaria shows no Omicron transmission before the end of November 2021

**DOI:** 10.1007/s15010-022-01767-1

**Published:** 2022-03-01

**Authors:** Jennifer Flechsler, Ute Eberle, Alexandra Dangel, Sabrina Hepner, Clara Wimmer, Johannes Lutmayr, Regina Konrad, Carola Berger, Laura Weise, Annika Sprenger, Jörg Zeitler, Natali Paravinja, Hildegard Angermeier, George Githure, Sandra Schmidt, Bianca Treis, Mercy Okeyo, Bernhard Liebl, Nikolaus Ackermann, Andreas Sing, Bavarian SARS-CoV-Public Health Laboratory Team, Christoph Baborka, Katja Bengs, Anja Berger, Volker Fingerle, Lorena Herrmann, Bernhard Hobmaier, Patrick Kudella, Verena Kukula

**Affiliations:** 1 Department of Public Health Microbiology, Bavarian Health and Food Safety Authority (LGL), Veterinärstraße 2, 85764 Oberschleißheim, Germany; 2 Unit of Virology, Bavarian Health and Food Safety Authority, Oberschleißheim, Germany; 3 NGS Core Unit, Bavarian Health and Food Safety Authority, Oberschleißheim, Germany; 4 State Institute of Health, Bavarian Health and Food Safety Authority, Oberschleißheim, Germany; 5 Ludwig Maximilians-Universität, Munich, Germany

**Keywords:** Variants of concern, Omicron, COVID-19, SARS-CoV-2, Surveillance

## Abstract

**Background:**

Five SARS-CoV-2 variants are currently considered as variants of concern (VOC). Omicron was declared a VOC at the end of November 2021. Based on different diagnostic methods, the occurrence of Omicron was reported by 52 countries worldwide on December 7 2021. First notified by South Africa with alarming reports on increasing infection rates, this new variant was soon suspected to replace the currently pre-dominating Delta variant leading to further infection waves worldwide.

**Methods:**

Using VOC PCR screening and Next Generation Sequencing (NGS) analysis of selected samples, we investigated the circulation of Omicron in the German federal state Bavaria. For this, we analyzed SARS-CoV-2 surveillance data from our laboratory generated from calendar week (CW) 01 to 49/2021.

**Results:**

So far, we have detected 69 Omicron cases in our laboratory from CW 47–49/2021 using RT-qPCR followed by melting curve analysis. The first 16 cases were analyzed by NGS and all were confirmed as Omicron.

**Conclusion:**

Our data strongly support no circulation of the new Omicron variant before CW 47/2021.

**Supplementary Information:**

The online version contains supplementary material available at 10.1007/s15010-022-01767-1.

## Introduction

Since the identification of SARS-CoV-2 on January 7 2020, and its spread into the whole world, five variants of the virus have been declared variants of concern (VOC). Having acquired genetic changes posing a risk to global health, the following variants and their respective phylogenic sublineages are presently categorized as VOC: B.1.1.7 (Alpha), B.1.351 (Beta), P.1 (Gamma), B.1.617.2 (Delta), and, since November 26th 2021, the variant B.1.1.529 (Omicron) [1]. So far, the occurrence of different variants coincided with different waves of infection. First detected in India in October 2020 and equipped with genetic mutations resulting in higher transmissibility than other SARS-CoV-2 variants, Delta is currently dominating the SARS-CoV-2 occurrence in most countries [2].

Based on data regarding transmissibility, risk of reinfection and effectiveness of vaccines [3], Omicron appears to have the potential to rapidly replace the Delta variant. Most interestingly, early studies from South Africa indicate a reduced risk for severe disease for Omicron infected patients compared to Delta infected patients, which might be due to population immunity acquired by infection and/or vaccination [4, 5]. However, large data volumes regarding Omicron are not yet available and are subject of current research [6]. Characteristic for Omicron is however, the vast number of mutations (approximately 30) in the spike protein of the virus. Some of these mutations and/or variant positions are already known from the Alpha, Beta, Gamma, and/or Delta variants (del69-70, K417N, E484A, P681H, N501Y, T95I, G142D/143–145del, T478K, N655Y, N679K) [2]. Among these mutations are amino acid substitutions that are connected with higher transmissibility (N501Y) or immune escape to antibodies (K417N) [7]. According to Sun et al., 2022, Omicron did not emerge from Delta, but branched off from the Gamma variant in mid-2020 [8]. Since then, Omicron might have circulated unnoticed in a small group of people or might have arisen in a chronically infected SARS-CoV-2 patient. Persistent SARS-CoV-2 infections have been suggested to trigger the emergence of new virus variants and are for instance observed in patients treated with immunosuppressive therapies and in patients suffering from hematological malignancies or advanced HIV [9]. Alternatively, Omicron might have developed in a non-human species, such as rodents and was lately re-transferred to humans [8]. The first detected/confirmed Omicron infection dates back to November 9, 2021 and, the WHO was informed about the new variant on November 24, 2021 by South Africa who recorded an increase of infection rates corresponding to the occurrence of Omicron [1]. By December 7, several hundred Omicron infections have been reported worldwide [10].

In our study, we investigated the circulation/transmission of Omicron in the German federal state Bavaria from calendar week (CW) 01/2021—49/2021 (January 1 2021–December 12 2021) by analyzing SARS-CoV-2 surveillance data from our laboratory. Based on these data, we conclude that Omicron was not distributed in Bavaria before CW 47/2021 (November 22–28 2021). In addition, we detected 69 Omicron cases in our laboratory in CW 47–49/2021 (November 22–December 12, 2021) using RT-qPCR followed by melting curve analysis and we were able to confirm 16 of these samples using Next Generation Sequencing (NGS; remaining samples were processed subsequently).

## Materials and methods

### Specimen collection and laboratory confirmation

Clinical respiratory specimens (nasopharyngeal swab samples) were stored in viral transport medium at 4 °C until further laboratory analysis at the Bavarian Health and Food Safety Authority in Oberschleissheim, Germany (LGL, Oberschleissheim, Germany).

### RT-qPCR (based on RNA extraction)

RT-qPCR and RNA extraction for SARS-CoV-2 diagnosis were performed on a Roche Cobas8800 (Roche Diagnostics, Switzerland), on a GeneXpert Infinity (Cepheid, Sunnyvale, USA), or on a MagExStar (Hamilton, Bonaduz, Switzerland). Roche Cobas8800 and GeneXpert Infinity are fully automated systems for RNA extraction and amplification, with integrated detection systems based on RT-qPCR. For SARS-CoV-2 diagnosis, we used the Xpert Xpress SARS-CoV-2 assay (Cepheid, Sunnyvale, USA) detecting the *N2 gene* and the *E gene* on the GeneXpert Infinity and the cobas® SARS-CoV-2 Test Kit detecting *orf1a/b gene* (SARS-CoV-2) and *E gene* (pan-Sarbecoviren) on the Cobas8800. When samples were analyzed on a MagExStar, RNA was extracted with an RNAdvance Kit from Beckman Coulter (Beckman Coulter, Indianapolis, USA) as previously described [11]. Detection of RNA was carried out by conventional RT-qPCR using the ampliCube Coronavirus SARS-CoV-2 assay from Mikrogen Diagnostik (Mikrogen, Neuried, Germany). RNA extraction and PCR assay detecting the envelope *(E) gene* of B-lineage betacoronavirus (FAM) and the *Orf1a gene* (HEX) were performed according to the manufacturer’s instructions. RT-qPCR was carried out on a Bio-Rad CFX96 Touch Real-Time PCR Detection System (Bio-Rad, Feldkirchen, Germany).

### VOC PCR and melting point analysis

Samples with a positive result for SARS-CoV-2 and a Ct-value < / = 35 were additionally screened for VOCs with spike protein deletion H69-V70 and the substitution mutations N501Y, L452R, and K417N using RT-qPCR followed by melting curve analysis. To identify VOCs, we used VirSNiP SARS-CoV-2 Spike del H69/V70, VirSNiP SARS-CoV-2 Spike N50iY, VirSNiP SARS-CoV-2 Spike L452R, and VirSNiP SARS-CoV-2 Spike K417N (TIB Molbiol, Berlin, Germany) combined with UltraPlex® 1-Step ToughMix® (4X) (Quanta bio, Beverly, USA) according to the manufacturer’s instructions. Depending on the current distribution of VOCs, we continuously adapted our diagnostic approach and, thus, we used different combinations of VirSNiP assays to distinguish the currently circulating mutants from each other. To differentiate between Alpha and Beta/Gamma, we used VirSNiP SARS-CoV-2 Spike del H69/V70 and VirSNiP SARS-CoV-2 Spike N501Y. To distinguish Alpha, Beta/Gamma and Delta we applied VirSNiP SARS-CoV-2 Spike del H69/V70, VirSNiP SARS-CoV-2 Spike N501Y and VirSNiP SARS-CoV-2 Spike L452R. For detection of Omicron, we currently use VirSNiP SARS-CoV-2 Spike L452R, VirSNiP SARS-CoV-2 Spike K417N and SARS-CoV-2 Spike del H69/V70. RT-qPCR was performed on a Bio-Rad CFX96 Touch Real-Time PCR Detection System (Bio-Rad, Feldkirchen, Germany).

### RT-qPCR data analysis

RT-qPCR data were evaluated with Bio-Rad CFX-Maestro software (version 1.1, 2019) or BioRad CFX96 Dx software (version 3.1, 2018). The amplification threshold was manually set within the exponential phase of the detection curve.

### Next generation sequencing and molecular surveillance of SARS-CoV-2

NGS-based molecular surveillance of SARS-CoV-2 was started in CW 02/2021. Samples, submitted for the entire diagnostic workflow of SARS-CoV-2 analysis (PCR, VOC-PCR and NGS) came from 75 different Bavarian administrative districts. Sequenced samples came from 73 of those districts, mainly submitted by the local health authorities for the entire diagnostic workflow. To a smaller extent, SARS-COV-2 positive RNA eluates were submitted from the same districts but from private laboratories providing PCR diagnostics but no NGS analysis.

Per week, we routinely sequenced a subset of SARS-CoV-2 positive samples determined by PCR with Ct values < / = 30. Samples with Ct values above 30 are not appropriate for our sequencing workflow. Samples for sequencing were primarily selected according to the following criteria: (1) samples with unclear VOC PCR results with Ct values < / = 30, and (2) PCR-based, clinical or epidemiological suspects for at time of analysis non-predominant VOCs ((B.1.1.7 (Alpha); B.1.351 (Beta); P.1 (Gamma) and B.1.617.2 (Delta) and sublineages). Additionally, capacities of up to 190 samples/week were filled with a preferably balanced set of samples from the different sample-submitting districts and outbreaks if outbreak information was available. Laboratory and bioinformatics NGS analysis of samples for generation of consensus genomes and classification of viral lineages was performed as described in [12]. For this study, the complete dataset was retrospectively reclassified with pangolin software [13, 14] version: 3.1.17, which reliably detects the Omicron variant (B.1.1.529 and sublineages BA*).

## Results

From CW 01/2021—CW 49/2021, 144,783 samples were examined for the presence of SARS-CoV-2 by RT-qPCR based methods at the LGL, Oberschleissheim, Germany. In total, 25,426 samples were tested positive for SARS-CoV-2 corresponding to a positive rate of 18%. Depending on Ct values, 21,441 of these samples were screened for SARS-CoV-2 VOCs by RT-qPCR followed by melting curve analysis, and 19.9% of SARS-CoV-2 positive samples (34.3% of samples with sequencing-suitable Ct values < / = 30) were analyzed by NGS. Based on VOC PCR screening we obtained the following results (see Table 1 and Supplementary Table 1).

**Table 1:** Number of VOCs detected at the LGL, Oberschleissheim, Germany by VOC PCR screening from CW 01/2021 to 49/2021 (for details on prevalence of different VOCs per calendar week, see Supplementary Table 1)

VOC	Number	CW
Alpha	9111	2–35 (January 11–September 5 2021)
Beta and gamma	553	3–30 (January 18–August 1 2021)
Delta	5612	17–49 (April 26–December 12 2021)
Omicron	69	47–49 (November 22–December 12 2021)

**Fig. 1:**
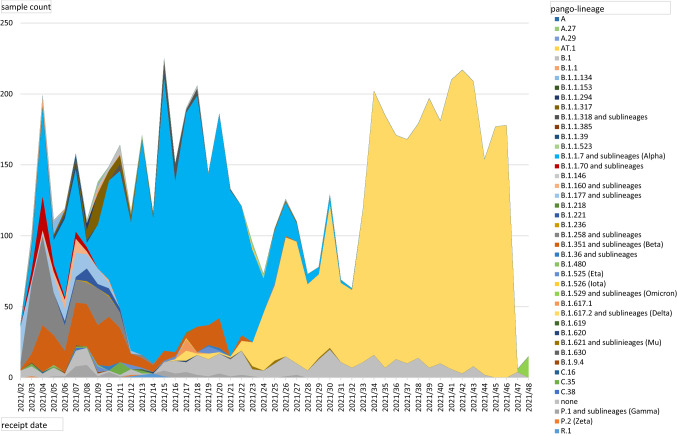
Viral lineages of NGS-analyzed samples. Viral lineages of all samples analyzed by whole genome sequencing in 2021 were retrospectively reclassified with pangolin version 3.1.17 (13, 14) and are shown based on receipt date (CW). VOC Omicron (light green) was not detected before week 47

Considering temporal distribution of detected VOCs and their occurrence per CW, we see a rapid rise of the Alpha variant starting in CW 02/2021, with a peak from CW 12–21/2021 and a last detected Alpha VOC in CW 35/2021. In parallel, we detected the variants Beta and Gamma from CW 03/2021—30/2021 with 553 samples in total. With a number of 9111 detected samples, the Alpha variant was clearly dominating the infection scenario those times. So far, we were able to identify 5612 Delta variants in our laboratory. From CW 17/2021, Delta lineages started replacing Alpha, Beta and Gamma lineages, and until now, Delta lineages are, according to our data the dominating SARS-CoV-2 species of the 4^th^ infection wave in the areas from which we obtained samples. Most interestingly, between CW 33–46/2021 we only detected variant Delta (B.1.617.2 and sublineages AY.*) by NGS analysis. Up to CW 49/2021, we recorded a total of 69 Omicron cases. Of these, 16 samples were analyzed by NGS and, Omicron could be confirmed in all 16 cases. Sequencing of the remaining 53 samples was still in progress when this study was performed. The first sample in which we detected the new Omicron variant by VOC PCR was obtained in CW47/2021, followed by 68 additional samples in CW 48–49/2021 (Fig. 1).

In addition, we retrospectively analyzed 146 samples from November 2021 by VOC PCR (spike protein mutation assays L452R, K417N and DEL69/70) that did not show a clear result in the L452R assay. According to our reanalyzed data, 49 of these samples were assigned to Delta lineages. Presumably, due to Ct-values > / = 35, the remaining 97 samples did still not show a distinct PCR result. However, we found that 64 of these samples did not have the substitution mutation K417N. Interestingly, 6 of these 97 samples turned out to belong to Delta sublineage AY.122 and had an additional SNP in the spike protein (T22928C → aa:S:F456L) located 11 bp downstream of mutation site L452R. This lead to a downshift of the melting curve in the L452R PCR assay in our samples (data confirmed by NGS). Only, 27 of the reanalyzed samples did not show results in PCR analysis due to low virus concentrations in samples. From this, we do not have any indication for the presence of Omicron and thus, we assume that no Omicron variants were included in these 146 samples.

The SARS-CoV-2 lineages of our samples, which we obtained by NGS, are mostly in accordance with results of VOC PCR screening (exceptions: SARS-CoV-2 samples having acquired mutations that are typically for another variant or that are located near spike protein mutation sites relevant for vPCR diagnostics). In 2021, 6537 positive SARS-CoV-2 samples have been analyzed by NGS in our department. For 6182 samples, sequencing was successful and SARS-CoV-2 lineages could be classified. Figure 1 outlines the development of prevalence of distinct virus lineages from CW 02–48/2021. For the purpose of clarity, we summarized the VOC and non-VOC lineages together with their respective sublineages, such as e.g. B.1.617.2 and AY.* for Delta (yellow), B.1.1.7 and B.1.1.7 + E484K or Q.1, Q.4 for Alpha (blue), or B.1.1.529/BA.1 for Omicron (light green). Consistent with the VOC PCR screening, Alpha variants were clearly dominating from CW 08–23/2021 with two smaller peaks of Beta variants between CW 03–12/2021 and CW 17–20/2021 and were almost completely replaced by Delta variants until CW 33/2021. In addition, we are able to show occurrence of first Omicron variants, labelled in light green, in CW 47/2021.

## Discussion

In this study, we analyzed VOC PCR data from CW 01-49/2021 and NGS data from CW 02-48/2021, generated at the LGL, Oberschleissheim, Germany. VOC PCR results were in congruence with NGS-based viral lineage classification. In total, we were able to sequence approximately 20% of SARS-CoV-2 positive samples in the analyzed time period with a median of 38% (60% based on samples with sequencing-suitable Ct values < / = 30) per CW. A major point of criticism during the last two years was the low SARS-CoV-2 sequencing surveillance in Germany and other countries in comparison to Denmark and the United Kingdom [15]. According to the gisaid database, Denmark and the United Kingdom sequenced and shared 26.9% and 11.2% of SARS-CoV-2 cases since January 10 2020, respectively. Within the same period, Germany sequenced and shared only 4.4% of SARS-CoV-2 cases [16]. By sequencing of 20% of SARS-CoV-2 positive samples, we exceeded this value. Furthermore, statistical models suggest that new variants can be detected by sequencing of 5% of SARS-COV-2 positive samples, given a prevalence of new variants of 0.1–1% [17]. Based on our sequencing rate together with the screening and sampling process, we assume that missing out upcoming lineages such as B.1.1.529 (Omicron) was prevented in our institute.

Our data show a clear dominance of the Alpha lineage among SARS-CoV-2 infections during the first half of the year and the replacement of Alpha by the Delta variant during the second half of the year, as observed in many countries around the world [18]. We reported the detection of the first Omicron variants in our department in CW 47/2021. Thus, from our results, we do not have an indication for Omicron transmission before declaration as VOC by the WHO at the end of November 2021.

Until CW 49, Delta was the most prevalent variant in Germany with a proportion of 96.5%. Until then, only several hundred Omicron cases had been reported in the European Union and European Economic Area. By CW 52, Omicron was already replacing Delta and became the pre-dominant variant in several European countries (i.e. proportion of Omicron cases in Denmark 92.3% and in France 70.5%). In Germany, the share of Omicron cases (29.7%) was still comparably low [19]. However, within the next few weeks, Omicron will also completely replace Delta in Germany. Thus, the rapid spread of Omicron across the world has taught us how valuable and important surveillance tools are.

The detection of this new SARS-CoV-2 variant by careful surveillance and the fast warning by the WHO helped us to take appropriate action, to investigate the new virus strain and to slow down the distribution of the variant [4, 20].

Limitations of the study are mainly due to technical restrictions: samples with Ct-values > / = 35 and > / = 30 are not suitable for VOC PCR and for whole genome sequencing, respectively. In addition, sampling might not have been representative depending on capacities and availability of laboratory infrastructure at local health authorities in different regions of Bavaria (i.e. sample submission to our lab only from 75 out of 96 districts during the analyzed period).

## Supplementary Information

Below is the link to the electronic supplementary material.

Supplementary file1 (DOCX 34 KB)

## Data Availability

Data available within the article or its supplementary materials.
