# Supplementary material for: Molecular SARS-CoV-2 surveillance in Bavaria shows no Omicron transmission before the end of November 2021
Source: Infection. 2022 Mar 1;50(3):761–6. doi: 10.1007/s15010-022-01767-1 (PMC8886714; doi:10.1007/s15010-022-01767-1)
Supplement: Supplementary file 1 — Supplementary file1 (DOCX 34 KB) [file 15010_2022_1767_MOESM1_ESM.docx]

**Molecular SARS-CoV-2 surveillance in Bavaria shows no Omicron transmission before end of November 2021**

Jennifer Flechsler^1,*^, Ute Eberle^2,*^, Alexandra Dangel^3,*^, Sabrina Hepner^3^, Clara Wimmer^2^, Johannes Lutmayr^2^, Regina Konrad^1^, Carola Berger^3^, Laura Weise^3^, Annika Sprenger^3^, Jörg Zeitler^2^, Natali Paravinja^2^, Hildegard Angermeier^2^, George Githure^2^, Sandra Schmidt^2^, Bianca Treis^2^, Mercy Okeyo^2^, Bernhard Liebl^4,5^, Nikolaus Ackermann^1,2,*^, Andreas Sing^1,4,5,*^ for the Bavarian SARS-CoV-Public Health Laboratory Team^6^

**Affiliations:**

^1^Dept. of Public Health Microbiology, Bavarian Health and Food Safety Authority

^2^Unit of Virology, Bavarian Health and Food Safety Authority

^3^NGS Core Unit, Bavarian Health and Food Safety Authority

^4^[State Institute of Health, Bavarian Health and Food Safety Authority, Oberschleißheim, Germany](https://www.eurosurveillance.org/search?option1=pub_affiliation&value1=State+Institute+of+Health%2C+Bavarian+Health+and+Food+Safety+Authority%2C+Oberschlei%C3%9Fheim%2C+Germany&option912=resultCategory&value912=ResearchPublicationContent)

^5^[Ludwig Maximilians-Universität, Munich, Germany](https://www.eurosurveillance.org/search?option1=pub_affiliation&value1=Ludwig+Maximilians-Universit%C3%A4t%2C+Munich%2C+Germany&option912=resultCategory&value912=ResearchPublicationContent)

*Contributed equally

**^6^Bavarian SARS-CoV-Public Health Laboratory Team**:

Christoph Baborka, Katja Bengs, Anja Berger, Volker Fingerle, Lorena Herrmann, Bernhard Hobmaier, Patrick Kudella, Verena Kukula

**Corresponding author:**

Prof. Dr. Dr. Andreas Sing, M.A. DTM&H

Bavarian Health and Food Safety Authority (LGL)

Veterinärstraße 2

85764 Oberschleißheim

[andreas.sing@lgl.bayern.de](mailto:andreas.sing@lgl.bayern.de)

Table S1: Results of SARS-CoV-2 PCR and vPCR screening by CW. Percentage values of vPCRs were calculated depending on SARS positve results
